# A mixed methods experience sampling study of a posttraumatic growth model for addiction recovery

**DOI:** 10.1038/s41598-024-53740-7

**Published:** 2024-02-21

**Authors:** Jason D. Runyan, Silas Vermilya, Megan St. Pierre, Nathan W. Brooks, Avery Fowler, Tia Brewer

**Affiliations:** 1 Indiana Wesleyan University, Marion, IN USA; 2 Hope House, Marion, IN USA

**Keywords:** Psychology, Human behaviour

## Abstract

Problematic substance use and addiction is a growing crisis in the United States. As a result, identifying factors that effectively promote addiction recovery is, currently, of particular societal importance. Informed by evidence that—while addiction can be perpetuated by stress-related impulsivity and decreased self-regulation—perceived social support is protective against addiction, we test a model for addiction recovery as a form of posttraumatic growth (PTG), focused specifically on close relationships and personal strength—two domains of PTG. In an initial study, we found that perceived social support and impulsivity predicted number of relapses in individuals in a substance use disorder recovery program. Using experience sampling, we then observed that experiencing a stressful event predicted impulsive behavior. However, experiencing closeness with others—a domain of PTG—was directly associated with perceived social support, and both predicted positive emotional states, which were, in turn, inversely associated with experiencing a stressful event. Further, when experiencing a stressful event, personal strength—also a domain of PTG—was inversely associated with impulsive behavior and was predicted by both perceived social support and positive emotional states. Finally, in a follow-up experiment, we found that an ecological momentary intervention targeting perceived social support decreased impulsivity and increased self-regulation—an aspect of personal strength—in a dose-dependent fashion. Taken together, our findings: (1) support a PTG model for recovery; (2) provide evidence for processes by which close supportive relationships are protective against addiction and relapse; and (3) indicate that self-regulation is responsive to a short in-the-moment perceived social support intervention. We suggest that these findings support the theory that addiction is a social disease in the sense that close personal interactions and supportive relationships: (a) buffer against stress-related impulsivity, thereby protecting against addiction and relapse; and (b) increase personal strength, thereby decreasing the probability of impulsive—including addictive—behavior and promoting recovery.

## Introduction

Over the past 20 years, problematic alcohol and drug use has been on the rise within the United States^1^. Drug overdose has risen to epidemic levels and continues to increase—an increase that sharpened during the COVID-19 pandemic^2^. In response, there has been a growing demand for substance use disorder (SUD) treatment programs and residential recovery homes. This high demand makes questions concerning factors which effectively promote addiction recovery of particular societal importance. In keeping with this, the Center for Disease Control (CDC) has recently stressed the need for timely data for meeting the demands of this growing crisis^2^. And, while psychological predictors of addictive behaviors, relapse, and substance use outcomes have been widely studied, the modeling of addiction recovery as a form of growth has only begun to receive attention and has yet to be studied within the daily life and experience of those in recovery.

### Addiction and growth in recovery

It has recently been suggested that coming to a deeper understanding of addiction recovery requires examining, not just relapse and abstinence, but growth in and through the recovery process^3,4^. To this effect, it has been theorized that posttraumatic growth provides a model for recovery. Posttraumatic growth has been defined as ‘positive psychological changes experienced as a result of the struggle with trauma or highly challenging situations’^5^. A little more descriptively, it refers to the process of adaptive dispositional change, which promotes well-being, continued psychological growth, and healthy relationships, following challenging and aversive events^6,7^. Posttraumatic growth has been divided into five domains: (1) appreciation of life; (2) relationship to others; (3) new possibilities; (4) personal strength, including an increased ability for self-regulation; and (5) spiritual change (see Table 1)^8^. To date, this process of change has been observed after numerous forms of traumatic or challenging events [e.g.,^9–16^].

**Table 1:** Domains of posttraumatic growth.

Domain	Dispositional growth
1. Appreciation of life	Increased appreciation of each day and moment, and of one’s life; and a change in one’s priorities concerning what is important
2. Relationship to others	A greater sense of closeness to others; increased likelihood of relying on others in times of need and acceptance of needing others; increased willingness to share one’s emotions and increased compassion for others; a greater effort in one’s relationships and appreciation for others
3. New possibilities	Increased awareness of new opportunities; developing new interests, a new path in life, and new purposes, and an increase likelihood of making changes to accomplish these
4. Personal strength	Increased ability to not be overwhelmed by difficult situation and for self-regulation; increased acceptance of outcomes and mental strength
5. Spiritual change	A greater understanding of spiritual matters, increased openness and authenticity about one’s own spirituality, and a stronger religious, or spiritual, faith

Consistent with a posttraumatic growth model of addiction recovery, addiction is known to be directly associated with stress and/or trauma [e.g.,^17–20^]. To add to this, the process of recovery itself can be seen as challenging and stressful [cf.^4^]. Further, it is well documented that addictive behaviors promote dependency at least in part by decreasing self-regulation such that engagement in these behaviors persist despite negative consequences [e.g.,^21^]. Continuation of these behaviors perpetuated by dependency, particularly as a way of coping with challenging and stressful events, and the ensuing intensification of negative consequences—including decreased self-regulation—results in a persistent cycle of stress and trauma. On a posttraumatic growth model, addiction recovery thus involves adaptive dispositional change, including increased self-regulation, which attenuates the continuation of these behaviors [cf.^4^].

Multiple studies have associated addiction recovery with change included as part of posttraumatic growth. These include spiritual change [e.g.,^22^], willingness to seek help [e.g.,^23^], and positive close relationships with others [e.g.,^24^]. However, only two studies have directly examined posttraumatic growth in relation to substance use and addiction recovery.

Foster et al*.*^25^ observed that, among undergraduate students in the United States, posttraumatic growth moderated a negative association between spirituality and religiosity, on the one hand, and drinking behavior, on the other. Additionally, Haroosh and Freedman^26^ found that, among individuals in addiction recovery programs in Israel, participation in a 12-step program was associated with increased abstinence as well as higher scores in the new possibilities and spiritual change domains of posttraumatic growth. Further, elevated levels of appreciation of life, personal strength, and spiritual change were observed among those who had served as a sponsor in 12-step programs.

### Impulsivity and addiction recovery

Consistent with models of addiction as involving decreased self-regulation, and with a posttraumatic growth model of recovery as involving increased self-regulation, multiple lines of research indicate that impulsivity is a relatively strong predictor of SUD and other behavioral addiction outcomes [cf.^27^]. To this end, the Urgency, Perseverance, Premeditation, Sensation Seeking, Positive Urgency Impulsive Behavior Scale (UPPS-P) has provided a useful model for impulsive tendencies as a multi-dimensional construct comprised of five factors: (1) negative urgency; (2) positive urgency; (3) lack of premeditation; (4) lack of perseverance; and (5) sensation seeking (see Supplementary Materials)^28–30^.

Like addiction, impulsive tendencies are directly associated with stress, and particularly early life stress [e.g.,^31^]. Additionally, a meta-analysis of research on impulsive tendencies and substance use outcomes revealed that these tendencies were associated with poorer substance use psychotherapy outcomes and decreased with SUD treatment [^32^; see also:^33,34^]. More recently, numerous studies have found associations between impulsive tendencies and substance use^35–37^.

### Perceived social support and addiction recovery

Additionally, consistent with the relationship to others domain of posttraumatic growth, many addiction treatment programs, such as 12-step programs, emphasize close supportive relationships as being central to recovery [cf.^26^]. In support of this emphasis, perceived social support—operationalized as having the sense of being cared for and of having people who are there for one in times of need^38,39^—has also been observed to be a predictor of substance use outcomes. For example, among individuals in recovery, perceived social support has been negatively correlated with relapse^40,41^, and positively correlated with less substance use^42^. Further, perceived social support has long been theorized to be protective against substance use problems^43,44^. In keeping with this theory, perceived social support has been observed to be negatively associated with substance use and addictive behaviors across multiple populations [e.g.,^45–50^].

### Experience sampling and addiction recovery

Experience sampling (ESM) and ecological momentary assessment (EMA) are methods involving repeatedly sampling physiological, behavioral, and/or experiential variables within the context of individuals’ daily lives [e.g.,^51,52^]. Since these methods involve measuring what is presently taking place—or has just taken place—they have the advantage of reducing biases that can arise when recall is removed from the time and context of what is being recalled. Additionally, these methods enable multilevel modeling (MLM) of within-person associations between variables as they fluctuate across the situations of daily life and over short periods of time (e.g., over the course of a day). In this way, they allow the modeling of changes that occur within individuals as they go about the day.

While research on smoking and smoking cessation served as an early application of these methods [e.g.,^53–55^], relatively few studies have used ESM or EMA to examine the daily life and experience of individuals with SUDs involving alcohol or illicit drugs [but see:^56–62^]. And most of these have focused on cravings as a predictor of substance use [e.g.,^63,64^].

A 2015 systematic review found that—up to that point—the majority of addiction-related EMA studies had focused on tobacco^65^. Additionally, a more recent review found that most studies have focused on associations between stress, affect, cravings, and substance use outcomes, but with mixed results regarding associations between negative affect and craving/substance use^66^. This being said, EMA studies have indicated that certain patterns of in the moment stress^67,68^, high positive affect^69^, and certain kinds of intense negative affect (i.e., anger, fear, and sadness)^70^, predict craving and substance use outcomes during SUD treatment.

### The present study

Despite the predictive value of self-regulation, impulsive tendencies, and perceived social support for recovery outcomes, there has yet to be an ESM or EMA study examining these constructs in the daily lives of those in recovery. Over the past 20 years, there has, however, been a considerable amount of research devoted to self-regulation, including several ESM studies [e.g.,^71–73^]. What has repeatedly been observed is that the ability to self-regulate can be developed as well as depleted [e.g.,^74–76^]. However, very little research has been done on interactions between self-regulation, impulsive tendencies, and perceived social support [cf.^77^]. Furthermore—as noted earlier—while posttraumatic growth has been proposed as a model for addiction recovery, only a couple of studies have examined posttraumatic growth in relation to recovery; and, here again, none have done so in daily life.

In the present study we, thus, examine self-regulation, impulsive behavior, and perceived social support, along with the relationship to others and personal strength domains of posttraumatic growth, using mixed methods—including experience sampling of the daily lives of individuals in SUD recovery.

In keeping with the theory that addiction recovery is a form of posttraumatic growth—and from evidence that, while addiction is in part perpetuated by decreased self-regulation and stress-related impulsive behavior, perceived social support may be protective against addiction—we developed a model consisting of the following variables:

- the experience of a stressful event;
- impulsive behavior;
- perceived social support;
- relationship to others (posttraumatic growth domain 2);
- positive emotional states; and
- personal strength, as a measure of self-regulation (posttraumatic growth domain 4).

**Figure 1:**
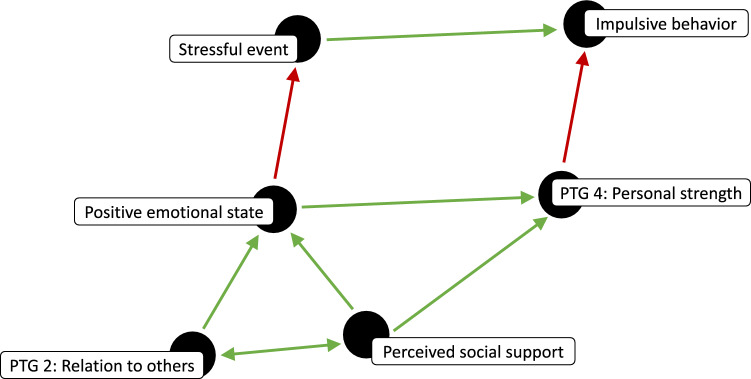
A directed acyclic graph^78^ for a model of addiction recovery. In the *top level*, experiencing a stressful event promotes impulsive behavior—including addictive behaviors. In the *middle level*, positive emotional states mitigate the experience of events as stressful and promotes personal strength, which in turn decreases impulsive behavior when experiencing a stressful event. In the *lower level*, perceived social support is synergistic with experiencing closeness to others, both of which promote positive emotional states, while perceived social support also promotes personal strength.

According to this model, experiencing a stressful event promotes impulsive behavior (see Fig. 1), which can include addictive behavior such as substance use. However, perceived social support buffers against impulsivity by promoting positive emotional states which mitigate the experience of events as stressful. Additionally, perceived social support is synergistic with experiencing closeness to others (posttraumatic growth domain 2) with whom one interacts, which also promotes positive emotional states. Further, even when experiencing a stressful event, personal strength (posttraumatic growth domain 4) decreases the probability of impulsive behavior, and perceived social support and positive emotional states promote personal strength.

Given this model, aspects of posttraumatic growth—namely, the experience of close supportive relationships (domain 2) and personal strength (domain 4)—are pivotal to addiction recovery. The theory is that experiencing close supportive relationships mitigates the experience of daily events as stressful, thereby reducing the frequency with which personal strength, and self-regulation, is taxed. Personal strength is, then, taxed less frequently. At the same time, personal strength is bolstered by experiencing close supportive relationships, and is needed and can be practiced when stressful events do arise in daily life. In this way, occurrences of impulsive behavior decrease as a result of the buffering context of experiencing close supportive relationships and the exercising of personal strength within this context.

To test this model for addiction recovery, we first examined associations between impulsive tendencies, perceived social support, and number of relapses in individuals currently in recovery for SUD. We then used ESM to investigate within-person associations between the variables in our model (see Fig. 1), as well as to examine daily stressors and situational factors. Finally, since associations do not provide an adequate basis for drawing causal inferences, we conducted a follow-up experiment to examine whether an ecological momentary intervention (EMI) targeting perceived social support might decrease impulsivity and increase self-regulation in a nonclinical sample.

## Methods

### Participants

All methods were approved by—and carried out in compliance with the guidelines and regulations of—Indiana Wesleyan University’s internal review board. In keeping with this, informed consent was obtained from all participants.

For the initial survey and ESM study, individuals in a SUD 12-step residential recovery program (N = 44) were recruited from within the county with the highest age-adjusted overdose death rate in Indiana (96.1;^79^). From the latest national data available at the time (2021), Indiana ranked 10^th^ in age-adjusted overdose death rates (43), and was part of a band of midwestern, southeastern, and northeastern states with some of the highest rates across the United States (including Ohio (48.1; 7^th^), Kentucky (55.6; 4^th^), Tennessee (56.6; 2^nd^), West Virginia (90.9; 1st), Pennsylvania (43.2; 9^th^), Maryland (42.8; 12^th^), and Delaware (54; 5^th^))^2^. Participants were incentivized by being informed that they would receive a $30 gift card for their time upon completion of the survey and 80% of the ESM sampling sessions. All participants were 18 years old or older, with an average age of 37.1 ± 8.66. Fifty-nine percent identified as male and 36.6% as female. Most participants were from Indiana (86.7%), and White/Caucasian (83.3%; Black or African American = 7.1%; Hispanic or Latino = 2.4%; Asian American = 2.4%; Other = 4.8%). On average, participants had been sober for 257.5 ± 232.3 (M ± SD) days. Forty-one (93.2%) participants completed all parts of the study.

For the experiment testing a perceived social support EMI, participants (N = 56) were recruited from several general education courses at Indiana Wesleyan University. Perspective participants (N = 100) were sent emails inviting them to participate in a psychological study as an option for receiving credit in their respective course. Upon expressing interest, participants were then given more information about the study, including that getting the maximum amount of credit required responding to at least 80% of the ESM sampling sessions. All participants were between the ages of 18 and 24. Twenty-five percent identified as male and 75% as female. Most participants were from the Midwest region of the United States (i.e., Indiana, Michigan, Ohio, Illinois, Wisconsin; 90.9%), and were White/Caucasian (93%; Black or African American = 5%; Asian American = 2%). Fifty participants (89.3%) completed the study.

### Materials

Qualtrics (qualtrics.com) was used to provide instructions, collect consent, and collect demographical data from all participants. Participants in recovery were given the UPPS-P to assess impulsive tendencies^28^ along with the Multidimensional Scale of Perceived Social Support (MSPP)^38^. They were also asked questions about their recovery, including how long they had currently been sober and whether they had relapsed since being in recovery and, if so, how many times.

LifeData (lifedatacorp.com) was used to administer all ESM and EMI notifications, prompts, and questions. Participants downloaded the LifeData mobile app on their Apple or Android device. ESM and EMI study protocols were then downloaded and delivered to participants through this mobile application on their own smartphones using the notification application native to their device’s operating system. Participants were able to see their response rate within the LifeData mobile app.

In the initial ESM study, each sampling session included a four item ESM version of the UPPS-P called the Momentary Impulse Scale, which asked participants to rate the extent to which they had engaged in impulsive behaviors over the past hour (see Supplementary Materials)^80^. It also included a question asking whether participants had had any social interactions over the past hour. If they said yes, they were then given the top-loading item from an ESM version of the posttraumatic growth inventory (PTGI) measuring the extent to which they had felt close to at least one person they had interacted with over the past hour^81^. This was used to measure the relation to others domain (domain 2) of posttraumatic growth. If they said no, participants were asked to rate the extent to which they had had a vivid imagination. This was simply to ensure that participants received the same number of questions regardless of how they answered. After this, participants were asked if they had experienced a stressful event in the last hour. If they said yes, they were given an open response question asking what caused the stress before being given the two top-loading items from the ESM version of the PTGI mentioned above measuring personal strength in relation to the stressful event they reported^80^. If they said no, participants were asked to briefly describe what they had been doing over the past hour, and to rate the extent to which they had enjoyed what they had been doing as well as the extent to which they had felt in control of their emotions. Here again, these questions were delivered to ensure participants answered the same number of questions regardless of how they responded.

In each session of the initial ESM study, participants were also asked to rate the extent to which they had experienced intense emotions as well as the extent to which these were positive over the past hour. Participants’ responses to these two items were averaged to provide a measure of the positivity and intensity of their emotional state. After this, participants were given the top-loading item from MSPSS, which was adapted for ESM to ask whether, over the past hour, they had had the sense that there was someone in their life who cared about their feelings. Finally, participants were asked where they currently were, given a list of options (i.e., outside, inside, work, residence home, room, car, other), and asked to check all that applied. When participants selected “other”, they were given a follow-up open response question asking them to specify where they were. They were also asked if they were currently with anyone.

In each session of the EMI experimental study, participants were given a 4-point Likert scale question asking them how much the statement that they had felt impulsive over the past hour reflected how they had felt; and a 4-point Likert scale question asking how much the statement that they had been good at resisting temptation over the past hour reflected their experience (see Supplementary Materials). These were used as face-value measures of impulsivity and self-regulation, respectively. But, here, the experimental group was first given a short perceived social support intervention asking them to list three people they knew who cared about them and how they were doing and feeling. They were, then, asked to spend a few minutes thinking about these people and the ways they support them. Alternatively, the control group received a prompt asking them to reflect on three events that occurred in their lives in the last day, and to spend a few minutes thinking about these events and how they made them feel.

R version 4.3.1 was used for MLM, and for calculating MLM effect sizes, using the lme4 and lmerTest packages to generate and test lmer models. Also, the ggdag package was used to generate directed acyclic graphs, and the pscl package was used to generate zero-inflated Poisson models. SPSS version 28.0.1.0 was used for all other statistical analysis.

### Procedure

In the initial study, participants were oriented to the study, and informed that they would receive a $30 gift card if they completed an online survey and at least 80% of the ESM sampling sessions. After informed consent, they were then given the survey. Surveys were administered at the participants’ addiction recovery residential home, using laptops that were provided for this purpose. The order of the UPPS-P and MSPSS was randomized to counterbalance against any order effects. Demographical and relapse questions were given at the end of the survey.

After completion of the survey, participants were instructed to download the LifeData mobile application onto their Apple or Android device. All participants had one or the other. Through the mobile application, participants were then instructed to download the study protocol, given a practice question, and informed that they would receive three study notifications a day for seven days at semi-randomized times: one in the morning (between 9am and 12 pm), one in the afternoon (between 1 and 4 pm), and one in the evening (between 5 and 8 pm). They were then shown how to respond to notifications, how to access and answer prompts and questions within the LifeData mobile app, and how to see their response rate. Finally, participants were given a phone number to use if they had any problems or questions.

At the end of ESM sampling, participants were given a $30 gift card if they had responded to at least 80% of the sampling sessions. All other participants were given a $20 gift card. A debriefing presentation was also given to participants which explained the central observations of the study and potential applications. Three participants were removed from the ESM portion of the study due to responding to three or less sampling sessions.

In the EMI experimental study, potential participants were notified through email about the option of participating in the study to receive points in their respective general education course. Those who replied and expressed interest were scheduled to come to the lab for orientation and to take a brief online survey to collect demographic data. As in our initial study, participants were then instructed to download the LifeData mobile application onto their Apple or Android device. Again, all participants had one or the other. Participants were randomly assigned to either the experimental or control group, and through a double-blind process were instructed to download either the experimental or control study protocol. Since the sampling schedule was kept the same as in the ESM study outlined above, participants were given the same instructions, and informed that they would need to respond to at least 80% of the sampling sessions to receive the maximum number of points possible for participation. They were, then, shown how to respond to notifications, how to access and answer prompts and questions within the LifeData mobile app, how to see their response rate, and given a phone number to use if they had any problems or questions.

At the end of the study, participants were sent an email debriefing them about the purpose of the study. If they had responded to at least 80% of the sampling sessions, they were also informed that they would receive the maximum points possible. All other participants were informed that they would still receive points, but not the full amount.

## Results

### Impulsive tendencies and perceived social support as predictors of number of relapses

We first wanted to examine the relationship between impulsive tendencies and perceived social support, and whether these variables predicted number of relapses.

There was a negative correlation between impulsive tendencies and perceived social support (r_37_ = − .374, *p* = .023), and both variables were significantly correlated with number of relapses (impulsive tendencies: r_36_ = .497, *p* = .002; perceived social support: r_37_ = − .392, *p* = .017). Likewise, single linear regression revealed that impulsive tendencies and perceived social support were independent predictors of number of relapses, accounting for 24.7% and 15.3% of the variability, respectively (see Table 2). However, when combined in a multiple linear regression model—while the overall model was significant (*F* = 5.455, *p* = .009) and explained 26% of the variance—only impulsive tendencies was a significant predictor. Consistent with the observed correlation between impulsive tendencies and perceived social support, this indicated multicollinearity between the two as predictors of number of relapses; i.e.—that the variance in number of relapses explained by impulsive tendencies and perceived social support was substantially shared. This also provided indication that impulsive tendencies mediated the inverse association between perceived social support and number of relapses.

**Table 2:** Linear regression models predicting number of relapses.

		B	SE	β	*t*	R^2^
Model 1	Impulsive tendencies	.019	.006	.497	3.337**	.247
Model 2	Perceived social support	− .054	.021	− .392	− 2.518*	.153
Model 3	Impulsive tendencies	.015	.007	.385	2.075*	
	Perceived social support	− .026	.026	− .184	− .994	.260

*p < .05 **p < .01.

Additionally, to account for a potential excess of participants without a relapse, we also ran a zero-inflated Poisson regression model. Again, impulsive tendencies (β(SE) = .011(.004), *t* = 2.498, *p* = .012) and perceived social support (β(SE) = − .045(.004), *t* = − 2.267, *p* = .023) were each independent predictors of number of relapses. However, when combined neither were significant (impulsive tendencies: β(SE) = .005(.006), *t* = .969, *p* = .332; perceived social support: β(SE) = − .031(.026), *t* = − 1.191, *p* = .234), again indicating multicollinearity between the two.

### Experience sampling

To further investigate our model for addiction recovery (see Fig. 1)—and, with it, the relationship between impulsivity and perceived social support—we then conducted an ESM study (N = 41). Participants were instructed to download an ESM mobile application onto their own smartphones. Through this application, they received three semi-random notifications per day for a week: one in the morning, afternoon, and evening. These notifications took them to questions asking about their experience and behavior over the past hour pertaining to the variables in our model of addiction recovery. We used MLM of participants’ responses to these questions to test within-person associations between these variables. Using ESM also enabled the specification and categorization of the kinds of stressors participants experienced throughout the day as well as whether there were situational factors associated with either experiencing a stressful event or impulsive behavior, on the one hand, or perceived social support and personal strength, on the other.

### Predictors of stressors within daily life

**Figure 2:**
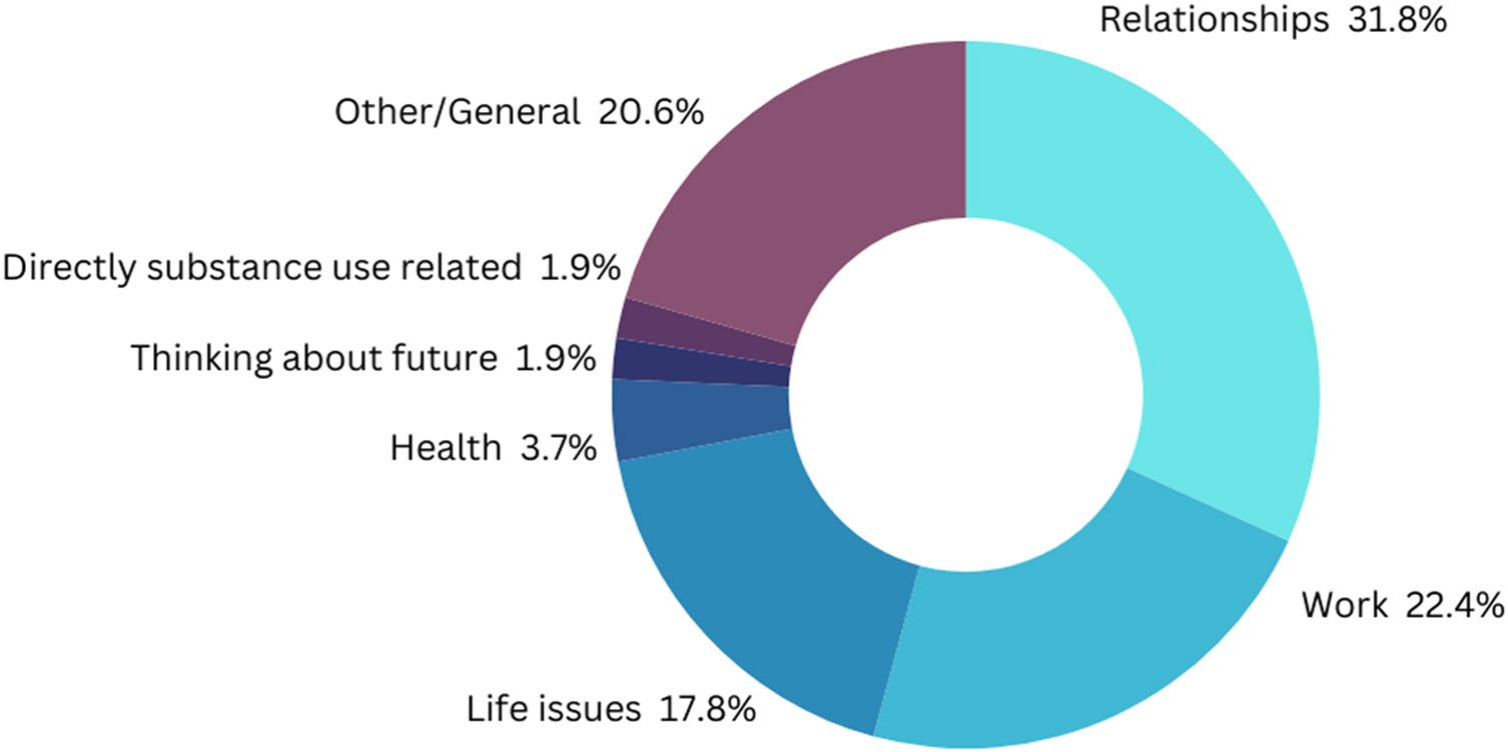
Reported momentary stressors.

According to our model, experiencing a stressful event promotes impulsive behavior. We, thus, asked our participants whether they had experienced a stressful event during each sampling session. On occasions where participants reported experiencing a stressful event, they were asked to specify the stressor. 671 total observations were made (response rate = 77.93%), and 107 momentary stressors were reported. Figure 2 displays the major categories of reported stressors. Relationship- (31.78%) and work- (22.43%) related stressors accounted for the majority. Of the major categories of stressors, those directly related to substance use were among the least frequently reported (1.87%; see Supplementary Materials).

MLM was used to assess within-person predictors of experiencing a stressful event. Participant was used as a random factor (y ~ x + (1|Participant)). In keeping with the observation that relationship- and work-related stressors were the most frequently reported, we found that experiencing a stressful event was directly associated with having interacted with another person (β(SE) =  .149(.047), *t* = 3.139, *p* = .002, r^2^ = .24; see Table 2), and being at work (β(SE) =  .073(.034), *t* = 2.139, *p* = .033, r^2^ = .23). At the same time, experiencing a stressful event was inversely associated with the positivity and intensity of one’s emotional state (β(SE) = − .0206(.004), *t* = − 5.255, *p* < .001, r^2^ = .11), as well as with perceived social support (β(SE) = − .024(.011), *t* = − 2.234, *p* = .026, r^2^ = .23).

Moreover—when used to predict the experience of a stressful event—there was a negative interaction between having interacted with another person and the positivity and intensity of one’s emotional state (Stressor ~ Interacted with another * Emotional state + (1|Participant); β(SE) = − .032(.0154), *t* = − 2.057, *p* < .05). Likewise, there was a negative interaction between having interacted with another person and perceived social support (Stressor ~ Interacted with another * Perceived social support + (1|Participant); β(SE) = − .057(.029), *t* = − 1.982, *p* < .05). This indicates that direct associations between experiencing a stressful event and having interacted with another person occurred when one’s positive emotional state as well as perceived social support were low.

Additionally, experiencing closeness to another person one had interacted with—posttraumatic growth domain 2 (PTG Relationship)—was trending toward an inverse association with experiencing a stressful event (β(SE) = − .023(.015), *t* = − 1.559, *p* = .119, see Table 3), and predicted perceived social support (β(SE) =  .568(.042), *t* = 13.63, *p* < .001, r^2^ = .53). Experiencing closeness to another also predicted the positivity and intensity of one’s emotional state (β(SE) =  .877(.131), *t* = 6.717, *p* < .001, r^2^ = .42), as did perceived social support (β(SE) =  .933(.098), *t* = 9.492, *p* < .001, r^2^ = .44). Further—when included as fixed factors in the same MLM (Emotional state ~ PTG Relationship + Perceived social support + (1|Participant))—both experiencing closeness to another (β(SE) =  .437(.148), *t* = 2.951, *p* = .003) and perceived social support (β(SE) =  .754(.126), *t* = 5.989, *p* < .001) remained significant predictors, indicating that they independently predicted the positivity and intensity of one’s emotional state.

**Table 3:** Within-person associations with experiencing a stressor, the positivity and intensity of one’s emotional state, and perceived social support.

y ~ x + (1|Partcipant)	Stressor (y)	Positivity + intensity of emotional state (y)	Perceived social support (y)
Interacted w/someone (x)	.149	.644(.421)	.816(.156)***
Positivity + intensity of emotional state (x)	− .0206(.004)***	–	.131(.014)
Perceived social support (x)	− .024(.011)	.933(.098)***	–
PTG relationship (x)	− .023(.015)	.877(.131)	.586(.042)***

*p < .05 **p < .01 ***p < .001.

**Figure 3:**
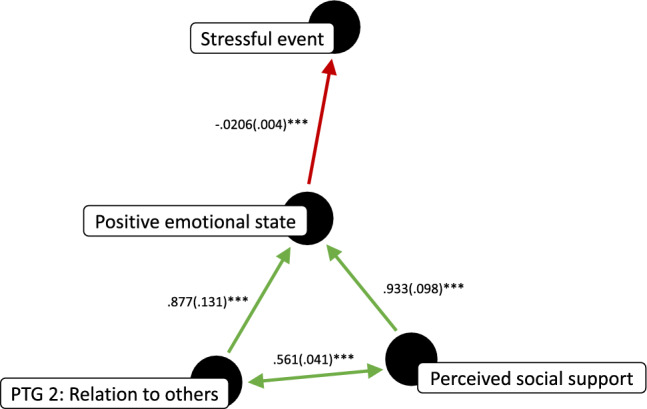
Summary of findings for experiencing a stressor as a directed acyclic graph. Unstandardized β*’*s for logistic regression coefficients are reported along with their respective standard errors.

Finally, to assess whether the positivity and intensity of one’s emotional state mediated the inverse association between perceived social support and experiencing a stressful event, we included both one’s emotional state and perceived social support as fixed factors (Stressor ~ Emotional state + Perceived social support), and tested whether perceived social support was still a significant predictor of experiencing a stressful event. While one’s emotional state remained a significant predictor (β(SE) = − .021(.004), *t* = − 4.796, *p* < .001), perceived social support did not (β(SE) =  .0007(.011), *t* = .063, *p* = .95), indicating that one’s emotional state mediated the inverse association between perceived social support and experiencing a stressful event (see Fig. 3).

### Predictors of impulsive behavior and personal strength

We also used MLM to assess within-person predictors of impulsive behavior and personal strength, again using participant as a random factor.

**Figure 4:**
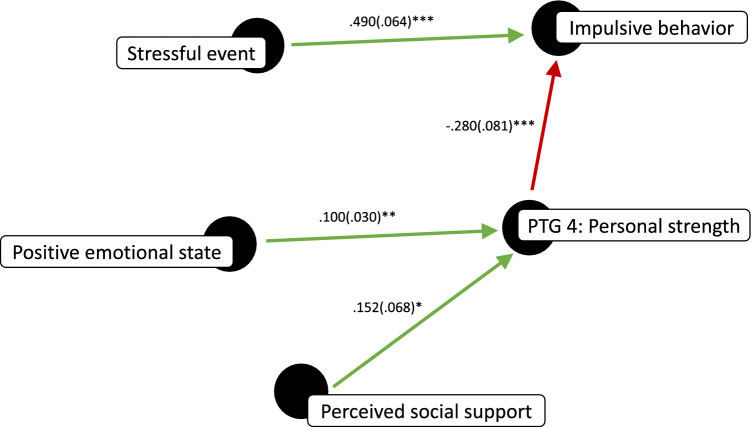
Summary of findings for impulsive behavior and personal strength as a directed acyclic *graph.* Unstandardized β*’*s for logistic regression coefficients are reported along with their respective standard errors.

Impulsive behavior was directly associated with experiencing a stressful event (β(SE) =  .490(.064), *t* = 7.64, *p* < .001, r^2^ = .57; see Table 4). Additionally, when experiencing a stressful event, impulsive behavior was inversely associated with experiencing personal strength (i.e., PTG Personal strength; β(SE) = − .280(.081), *t* = − 3.456, *p* < .001, r^2^ = .52)—posttraumatic growth domain 4—and with the positivity and intensity of one’s emotional state (β(SE) = − .021(.008), *t* = − 2.725, *p* = .007, r^2^ = .54). Further, personal strength was directly associated with both perceived social support (β(SE) =  .152(.068), *t* = 2.22, *p* = .029, r^2^ = .61), and the positivity and intensity of one’s emotional state (β(SE) =  .100(.030), *t* = 3.382, *p* = .001, r^2^ = .48). And, when both experiencing personal strength and the positivity and intensity of one’s emotional state were included as fixed factors, only the former was a significant predictor of impulsive behavior (Impulsive behavior ~ PTG Personal strength + Emotional state + (1|Participant); PTG Personal strength: β(SE) = − .240(.099), *t* = − 2.413, *p* = .019; Emotional state: β(SE) =  .004(.027), *t* = .151, *p* = .881). This indicated that personal strength mediated the negative association between the positivity and intensity of one’s emotional state and impulsive behavior (see Fig. 4).

**Table 4:** Within-person associations with impulsive behavior and the personal strength domain of post traumatic growth (PTG).

y ~ x + (1|Partcipant)	Impulsive behavior (y)	PTG personal strength (y)
Stressor (x)	.490(.064)***	–
PTG personal strength (x)	− .280(.081)***	–
Positivity + intensity of emotional state (x)	− .021(.008)**	.100(.30)**
Perceived social support (x)	− .023(.020)	.152(.068)*

*p < .05 **p < .01 ***p < .001.

### Predictors of closeness to others, perceived social support, & positive emotions

We, further, used MLM to examine situational predictors of being with others, perceived social support, and experiencing closeness to others.

Being with another person at the time of reporting predicted experiencing closeness to another person one had interacted with (i.e., PTG Relationship; β(SE) =  .391(.088), *t* = 4.46, *p* < .001, r^2^ = .373; see Table 5), as well as perceived social support (β(SE) =  .325(.097), *t* = 3.349, *p* < .001, r^2^ = .44), and the positivity and intensity of one’s emotional state (β(SE) =  .600(.269), *t* = 2.230, *p* = .026, r^2^ = .385). Additionally, when experiencing closeness to another, perceived social support, and the intensity and positivity of one’s emotional state were each included as fixed factors in an MLM predicting being with another person (With another person ~ PTG Relationship + Perceived social support + Emotional state + (1|Participant)), only experiencing closeness was a significant predictor (β(SE) =  .092(.021), *t* = 4.352, *p* < .001). This indicated that experiencing closeness mediated the associations between being with another person and perceived social support (β(SE) = − .019(.018), t = − 1.072, *p* = .284), as well as between being with another person and the intensity and positivity of one’s emotional state (β(SE) =  .002(.006), *t* = .308, *p* = .758).

**Table 5:** Within-person situational associations with experiencing closeness to another (PTG Relationship), perceived social support, and positive emotional states.

y ~ x + (1|Partcipant)	Closeness to another (y)	Perceived social support (y)	Positivity + intensity of emotional state (y)
Outside (x)	.022(.112)	− .146(.131)	− .134(.357)
Inside (x)	.018(.099)	.278(.115)*	.285(.314)
Work (x)	− .339(.96)***	− .456(.113)***	− .609(.315) ^^^
Residence home (x)	− .067(.91)	.123(.104)	.309(.285)
Room (x)	− .049(.134)	− .074(.141)	.140(.398)
Car (x)	.154(.117)	− .081(.135)	.308(.367)
With another person (x)	.391(.88)***	.325(.97)***	.600(.269)***
Other (x)	.248(.117)*	.541(.138)***	.659(.389)^^^

^*p* < .1 *p < .05 ***p < .001.

We also found that being at work was inversely associated with experiencing closeness to another (β(SE) = − .339(.096), *t* = − 3.516, *p* < .001, r^2^ = .365), as well as with perceived social support (β(SE) = − .456(.113), *t* = − 4.014, *p* < .001, r^2^ = .446). However, when both were included as fixed factors in an MLM predicting being at work (Being at work ~ Perceived social support + PTG Relationship + (1|Participant)), only perceived social support was a significant predictor (β(SE) = − .054(.015), *t* = − 3.465, *p* < .001), indicating that perceived social support mediated the inverse association between being at work and experiencing closeness (β(SE) = − .028(.019), *t* = − 1.479, *p* = .140).

Finally, experiencing closeness to another (β(SE) =  .248(.117), *t* = 2.113, *p* = .035, r^2^ = .359), and perceived social support (β(SE) =  .541(.138), *t* = 3.929, *p* < .001, r^2^ = .447), were directly associated with participants selecting the ‘Other’ option when reporting where they were. Here again, when both were included as fixed factors (Other ~ Perceived social support + PTG Relationship + (1|Participant)), only perceived social support was a significant predictor (β(SE) =  .023(.0132), *t* = 2.26, *p* < .05), indicating that it also mediated the inverse association between selecting ‘Other’ and experiencing closeness (β(SE) =  .005(.016), *t* = .348, *p* = .728).

**Figure 5:**
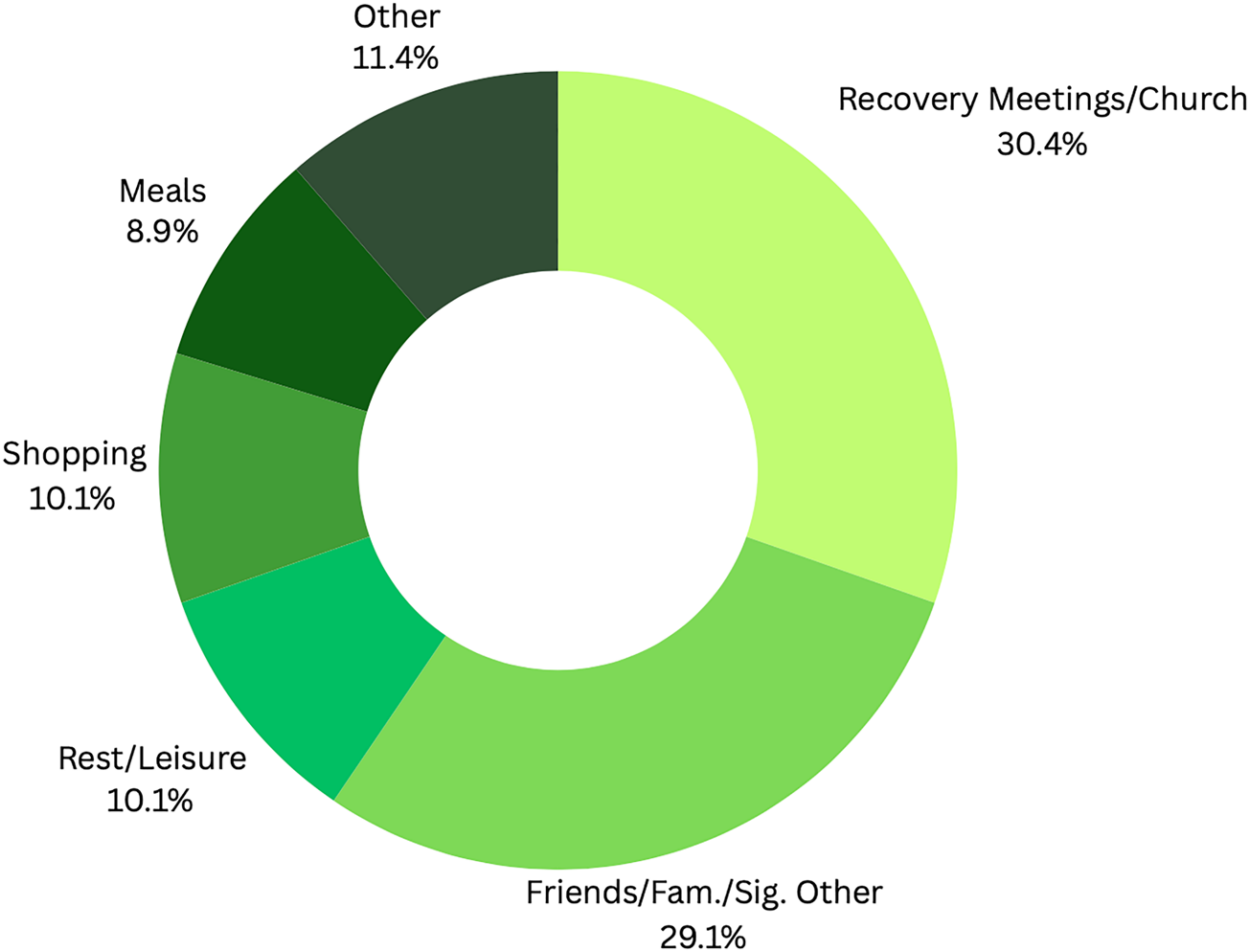
Specified ‘Other’ situational factors.

After selecting the ‘Other’ option (n = 79), participants were asked to specify where they were. Figure 5 provides the percentages of the major categories of reported situational factors. Being at a recovery meeting/church service or being with family, friends, or significant others accounted for 59.49% of these situational factors. Resting or engaging in leisure activities, shopping, or eating meals with others accounted for another 29.11% of these factors (see Supplementary Materials).

### A perceived social support intervention decreases impulsivity & promotes self-regulation

Central to our model is the theory that perceived social support bolsters self-regulation, and is, in this way, protective against impulsivity. To test this, we performed a follow-up experiment wherein a nonclinical sample was randomly assigned to either a control (n = 21) or an experimental group (n = 29)—the latter of which repeatedly received a smartphone-based EMI targeting perceived social support. We then used ESM to examine within-person impulsivity and self-regulation at three semi-random times: once in the morning, afternoon, and evening. 958 observations were made (response rate = 91.24%).

Consistent with previous studies indicating that self-regulation can be depleted throughout the day [e.g.,^82–87^], we found that, from morning to evening, within-person impulsivity increased (impulsivity ~ sampling time + (1|Participant); β(SE) =  .164(.036), *t* = 4.61, *p* < .001, r^2^ = .368), and self-regulation decreased (Self-regulation ~ Sampling time + (1|Participant); β(SE) = − .077(.035), *t* = − 2.26, *p* = .026, r^2^ = .308). Additionally, impulsivity and self-regulation were inversely associated (Impulsivity ~ Self-regulation + (1|Participant); β(SE) = − .364(.038), *t* = − 9.67, *p* < .001, r^2^ = .404). Within the control group, neither within-person impulsivity (Impulsivity ~ Session number + (1|Participant); β(SE) =  .460(.380), *t* = − 1.209, *p* = .228) nor self-regulation (Self-regulation ~ Session number + (1|Participant); β(SE) = − .091(.427), *t* = − .213, *p* = .832) changed across EMI sessions. However, within individuals in the experimental group, impulsivity decreased (β(SE) = − .036(.008), *t* = − 4.80, *p* < .001, r^2^ = .356), and self-regulation increased (β(SE) =  .023(.007), *t* = 3.54, *p* < .001, r^2^ = .365), across EMI sessions. This implies that there was a dose dependency effect within individuals receiving the perceived social support intervention.

## Discussion

Informed by evidence that, while addiction is perpetuated by decreased self-regulation and stress-related impulsive tendencies, perceived social support is protective against addiction and relapse, we developed a model for addiction recovery as a form of posttraumatic growth (see Fig. 1). According to this model, experiencing close supportive relationships increases positive emotional states, thereby mitigating the experience of stressful events, and thus the frequency with which personal strength, and self-regulation, is taxed. Additionally, experiencing close supportive relationships and positive emotional states promote personal strength, which decreases the probability of stress-related impulsive, including addictive, behavior.

**Figure 6:**
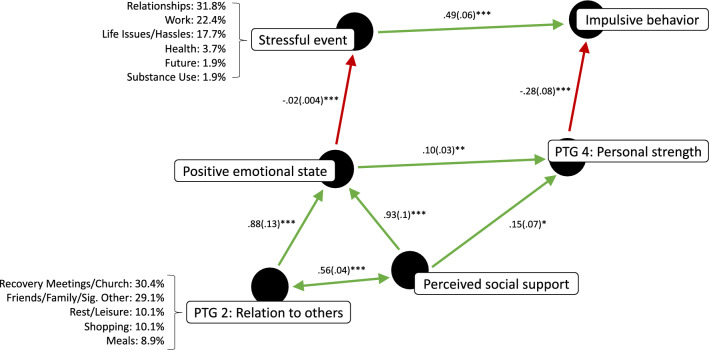
Summary of multilevel modeling of ESM data as a directed acyclic graph. Unstandardized β*’*s for logistic regression coefficients are reported along with their respective standard errors. Also included are the most frequently reported stressors as well as the situational factors directly associated with experiencing closeness to another person and perceived social support. ***p < .001 **p < .01 *p < .05.

In support of this model, we found that impulsive tendencies and perceived social support were inversely related, and that each predicted number of relapses amongst residence of a SUD recovery program. Further, using ESM, we found that experiencing a stressful event was directly associated with impulsive behavior, but inversely associated with positive emotional states, which were, in turn, directly associated with perceived social support and experiencing closeness to another. We also observed that, when an individual was experiencing a stressful event, personal strength—which was directly associated with both perceived social support and positive emotional states—was inversely associated with impulsive behavior (see Fig. 6).

Interestingly, relationships with friends, family, children, romantic partners, and other personal relations, were the most commonly reported stressors (31.78%), followed by work (22.43%; see Fig. 2 and Table 3). Also, having interacted with another person in the past hour was directly associated with experiencing a stressful event; but this association was moderated by low positive emotional states and perceived social support. Conversely, being with another person at the time of reporting was directly associated with experiencing closeness to another, perceived social support, and positive emotional states. And experiencing closeness to another mediated the association between being with another person and perceived social support as well as between being with another person and positive emotional states. Further, unguided self-reports revealed that experiencing closeness to another and perceived social support were directly associated with being in recovery meetings or church meetings, being with friends, family, and/or significant others, resting or having leisure time, and/or with having meals with friends or family (see Fig. 5).

So—on the one hand—personal relationships were the most frequently reported source of stress. And personal interactions were associated with experiencing a stressful event when positive emotional states and perceived social support were low. But—on the other hand—personal interactions were associated with experiencing relational closeness. And this closeness, in turn, mediated associations between personal interactions and perceived social support, as well as between personal interactions and positive emotional states. Further, experiencing this closeness was associated with being in social gatherings characteristically aimed at being supportive (i.e., recovery meetings and church meetings), as well as with close others or resting.

Because the observed associations alone do not provide an adequate basis for drawing causal inferences, we performed an experiment to examine whether perceived social support might buffer against impulsivity and boost self-regulation in daily life—a central tenet of our model. The multicollinearity we observed between perceived social support and impulsive tendencies in predicting relapse suggested this might be the case. But, to test this directly, we used a two-group experimental design using a nonclinical sample. Individuals in the experimental group repeatedly received a brief perceived social support intervention via their smartphones over the course of a week and exhibited decreased impulsivity and increased self-regulation in a dose-dependent manner. This pattern, however, was not observed within the control group. Within the context of our other observations, this implies that perceived social support is amenable to intervention and can buffer against impulsivity, while promoting self-regulation throughout the day. This would explain the multicollinearity we observed between perceived social support and impulsive tendencies in predicting relapse. It also provides an explanation as to how perceived social support can be protective against addiction and relapse [e.g.,^43,44^]. However, more work needs to be done to elucidate this process.

Nevertheless, our findings provide evidence that addiction is a social disease in the sense that close personal interactions and supportive relationships:

- buffer against stress-related impulsivity, thereby protecting against addiction and relapse; and
- promote personal strength, including self-regulation, thereby decreasing the probability of impulsive—including addictive—behavior and promoting recovery.

Given this, close supportive relationships and personal strength—two domains of posttraumatic growth—are pivotal to addiction prevention and recovery.

In addition to our findings here, converging lines of research support this theory. First, it has been observed that general social support predicts abstinence-specific self-efficacy^88^. Second, active participation in 12-step programs—which are focused on forming close supportive relationships and, within this context, growing and gaining personal strength—predicts better outcomes among those with alcohol use disorder [e.g.,^24,89^]. And third, while opioid-involved overdose deaths rose steadily in the United States from 21,089 in 2010 to 47,600 in 2017, there was a dramatic jump during COVID-19 isolation to 68,630 in 2020, and then 80,411 in 2021^2^. There was, thus, a 44% increase from 2017 to 2020, and an additional 17% increase between from 2020 to 2021, during and shortly after COVID-19 isolation.

Taken together, these observations provide evidence that the escalating substance use crisis in the United States may be, to a significant degree, the result of increasing relational isolation—in the sense of a lack of close supportive relationships—within the context of increased access to highly addictive opioids. While confirmation of this theory would require more direct research, it helps make sense of several developments in recovery research.

There has been growing empirical work on the concept of recovery capital^90,91^. The notion is that four domains of resources or skills are instrumental in recovery, two of which are social and personal (or human). Consistent with this idea, a systematic review of empirically based work on recovery has yielded evidence for five recovery processes involving (1) connectedness, (2) hope and optimism about the future, (3) changes in identity, (4) meaning in life, and (5) empowerment^92^. Both evidence to the effect that social and personal resources and skills are instrumental in recovery, and evidence that recovery involves connectedness and empowerment, align with recovery requiring close supportive relationships and personal strength. It also aligns with the theory that addiction is a social disease precipitated, in part, by isolation. Further, these theories—along with evidence that recovery involves changes in identity—align with ongoing work to the effect that addiction recovery is a process of undergoing an identity change due to changes in social networks and activities, whereby socially mediated social/relational and personal abilities develop^93,94^.

More broadly, it bears mentioning that our findings concerning personal strength and self-regulation are consistent with research indicating that self-regulation is a limited resource which can be developed and bolstered. Numerous studies indicate that, like caloric energy or physical strength, self-regulation can be depleted by, among other things, coping with stress [e.g.,^72,74,76,95–97^]. Consistent with this, we observed that self-regulation decreased throughout the day. However, there is also evidence that self-regulation can be strengthened through exercise or practice [e.g.,^74,75,98^], and that one’s beliefs, or perceptions, can affect self-regulation depletion [e.g.,^99^]. To add to these observations, we observed that increased perceived social support was associated with increased personal strength, and that a perceived social support intervention boosted self-regulation, in daily life. But what’s more, we also observed that perceived social support, along with experiencing closeness to another person, was associated with increased positive emotional states and a decrease in the likelihood of experiencing an event as stressful.

Though more research on these interactions is needed—when taken together—our findings imply that perceived social support and close personal interactions promote self-regulation, while at the same time buffering against stressors and thus reducing the need to use it. And this implication is not just consistent with previous research on self-regulation, but also with work on perceived social support as protective against addiction and relapse [e.g.,^43,44,100^]. In this vein, our findings offer two ways that perceived social support can have this protective effect:

1. by buffering against stress and reducing the need to use self-regulation; and
2. by boosting self-regulation.

Our observations, thus, connect previous findings on self-regulation and perceived social support, thereby uncovering a promising approach to strengthening self-regulation and a promising explanation as to how perceived social support can be protective.

It also bears mentioning that our findings—and the model for addiction recovery they support—fit with Fredrickson’s broaden-and-build theory concerning how positive emotions promote the expansion of one’s thinking and behavior in healthy ways [e.g.,^101–103^]. According to this theory, positive emotions open individuals’ agential capacities, thereby increasing the scope of what they are able to pay attention to, think about, and do (i.e., their ‘attention and thought-action repertoires’), beyond stress-related tendencies or impulses. The expression of these broadened capacities helps individuals develop enduring personal resources, including physical and intellectual to social and relational resources.

Our ESM findings suggest that, at least in part, the way perceived social support decreases impulsivity and increases self-regulation, is by increasing positive emotional states. And, as a result, in these instances the probability increases that one will engage in behavior other than impulsive behavior. In this way—consistent with the broaden-and-build theory—one’s thought-action repertoire is broadened beyond impulsive behavior on these occasions.

**Figure 7:**
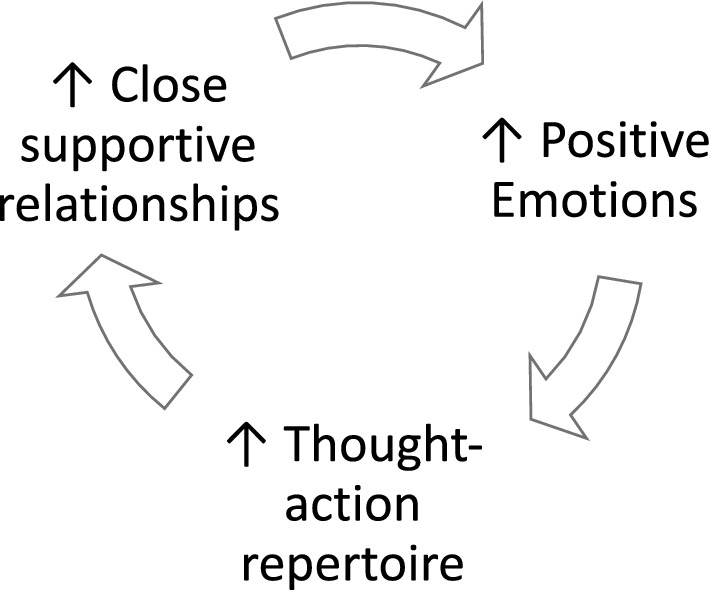
Theoretical synergistic ‘broaden and build’ feedback loop. In this loop, positive emotions ‘broaden’ a person’s thought-action repertoire beyond impulsive tendencies. This promotes the development of close supportive relationships as a personal resource which, in turn, promotes positive emotions.

Again, further experimentation is required to elucidate the specifics of the processes by which perceived social support might affect impulsive behavior. This would include defining the nature of the involved emotional states. However, it is worth noting that our findings indicate that relational resources (e.g., close personal interactions and supportive relationships) might, in turn, help ‘broaden and build’ individuals’ positive emotions—a possibility which, within the context of addiction recovery, warrants further study. Thus, close supportive relationships might complete a synergistic feedback loop for the expansion of personal resources (see Fig. 7).

While this study has multiple limitations, as the first investigation of a posttraumatic growth model of recovery focused on close supportive relationships and personal strength, it opens several avenues for future study into the nature of addiction and recovery.

Here, we provide evidence that two domains of posttraumatic growth—relationship to others and personal strength—are associated with decreased impulsive behavior, which in turn predicts number of relapses in a sample of individuals in SUD recovery. However, further work is needed to explore other domains of posttraumatic growth and examine the effects of interventions targeting these domains [cf.^4^]. Additionally, further experimental investigation is needed to tease out the causal processes by which the perceived social support intervention used here decreased impulsivity and promoted self-regulation; and, thus, to test the causal processes suggested by our ESM findings. Here, we simply provide initial evidence that experiencing close supportive relationships, in part by increasing positive emotional states, decreases stress-related impulsivity and increases personal strength. Further, our finds are largely associative, and we only show a causal effect in a non-clinical population. Thus, a substantial amount of work—which will need to rely more heavily on experimentation—remains to replicate and further define this process, and to test its generalizability, in both clinical and non-clinical populations. Moreover, our sample size was relatively small, bringing up questions about the power of our study.

Finally, our findings present the possibility that the processes in question—i.e., involving the experience of close supportive relationships, emotional states, and the exercise of personal strength—may hold significance beyond addiction recovery. These processes may have a broader role in facilitating psychological growth through traumatic or challenging situations when impulsive inclinations are high. In other words, they may be general processes of posttraumatic growth. But this too will require further study. While we focused on individuals in SUD recovery within an area of the United States with a high age-adjusted overdose death rate, additional research is required to ascertain the generalizability of our findings, and to further develop and evaluate interventions for impulsivity and self-regulation targeting perceived social support.

### Supplementary Information

Supplementary Information.

## Data Availability

The datasets used and analyzed during the current study are available from the corresponding author upon reasonable request.
