# Author Correction: A mixed methods experience sampling study of a posttraumatic growth model for addiction recovery

**DOI:** 10.1038/s41598-024-56471-x

**Published:** 2024-03-13

**Authors:** Jason D. Runyan, Silas Vermilya, Megan St. Pierre, Nathan W. Brooks, Avery Fowler, Tia Brewer

**Affiliations:** 1 Indiana Wesleyan University, Marion, IN USA; 2 Hope House, Marion, IN USA

Correction to: *Scientific Reports* 10.1038/s41598-024-53740-7, published online 21 February 2024

The Acknowledgments section in the original version of this Article was incomplete.

“This research was funded by an endowed grant funded by the Lilly Foundation. The authors wish to acknowledge Cole Marvin, Nathan Woodard, Tomecio Hardy, Reka Brooks, Zakari Smith, Caleb Heck, Corey Griffin, Jake Lively, Cameron Sarin, and Peyton Bollhorst for their assistance with study design, participant recruitment, and/or manuscript editing. Additionally, the authors would like to thank the individuals in recovery who allowed us to learn from their experience. The authors would also like to thank Dawn W. Foster for valuable suggestions and insights regarding study design, and Timothy A. Steenbergh for valuable suggestions and insights regarding design and data analysis.”

now reads:

“This research was funded by an endowed grant from the Lilly Foundation. The authors wish to acknowledge Isaac Alsup, Cole Marvin, Nathan Woodard, Tomecio Hardy, Alivia Beaver, Reka Brooks, Zakari Smith, Caleb Heck, Corey Griffin, Jake Lively, Cameron Sarin, and Peyton Bollhorst for their assistance with study design, participant recruitment, and/or manuscript editing. Additionally, the authors would like to thank the individuals in recovery who allowed us to learn from their experience. The authors would also like to thank Dawn W. Foster for valuable suggestions and insights regarding study design, and Timothy A. Steenbergh for valuable suggestions and insights regarding design and data analysis.”

The original Article has been corrected.

