# Supplementary material for: A mixed methods experience sampling study of a posttraumatic growth model for addiction recovery
Source: Sci Rep. 2024 Feb 21;14:3511. doi: 10.1038/s41598-024-53740-7 (PMC10881473; doi:10.1038/s41598-024-53740-7)

Addiction recovery in daily life: Recovery as a form of personal growth in close supportive relationships

Jason D. Runyan,[1]* Silas Vermilya,[1] Megan St. Pierre,[1] Nathan W. Brooks,[1] Avery Fowler,[1] Tia Brewer[2]

[1] Indiana Wesleyan University, Marion, IN
[2] Hope House, Marion, IN
* Corresponding author: Jason.runyan@indwes.edu

Supplementary Materials

Table S1. Category and subcategory frequencies of momentary stressors (n=107).

RELATIONSHIPS: 34 (31.78%)

- Relationship with specific individuals: 18
  - Ex's and/or other parent of children: 7
  - Kids: 5
  - Oneself: 1
- Helping others or people asking for help: 5
- Residence house relationships: 4
- Friends leaving house/relapse: 2
- Talking to friends about going to rehab: 2
- Sexual or romantic relationships: 2

WORK: 24 (22.43%)

- Work in general: 15
- Equipment, tool, or resource issue: 3
- New on the job: 3
  - Physical & drug test for new job: 1
- Sick at work: 1
- Boss: 1
- Stress leading to leaving work: 1

LIFE ISSUES/HASSLES: 19 (17.76%)

- Disciplinary consequences: 5
  - DCS paperwork: 1
  - Court/Probation hearing: 1
  - Anger management class: 1
- Store: 2
- Trying to do better at working hard and prioritizing: 1
- Phone issues: 2
- Car issues: 1
- Driving: 2
- Bills: 1
- Arranging living space: 1
- Going out to eat: 1
- Cold weather: 1

HEALTH: 4 (3.74%)

- Oneself: 3
- Family: 1

THINKING ABOUT THE FUTURE: 2 (1.87%)

- Worries about the future: 1
- Leaving residency and being on own: 1

ISSUES DIRECTLY RELATED TO SUBSTANCE USE: 2 (1.87%)

Handling issues sober: 1
Cravings: 1

GENERAL/OTHER: 22 (20.56%)

'Spiritual matters': 13
'Worldly matters': 3
Thoughts: 2
'A personal experience': 1
Mental stress: 1
The survey: 1
Setting up a new ministry: 1

Table S2. Situational factors specified under 'Other' (n=79).

RECOVERY MEETINGS/CHURCH SERVICE: 24 (30.38%)

- Church: 11
- Recovery meeting: 6
- Recovery worship service: 4
- Bible study/devotional: 2
- Class: 1

FRIENDS/FAMILY/SIGNIFICANT OTHER: 23 (29.11%)

- Friend's house: 9
- With family: 7
  - Extended family's house/visiting family: 5
  - With kids: 2
- With Significant others': 7
  - Significant other's house: 3
  - Wife: 2
  - With significant other and daughter: 2

REST/LEISURE: 8 (10.13%)

- Break room at work: 3
- Exercising: 2
- Meditation: 1
- Couch: 1
- Watching TV: 1

SHOPPING: 8 (10.13%)

MEALS: 7 (8.86%)

- 'Friendsgiving meal': 1

OTHER: 9 (11.39%)

- Community service: 1
- Job interview: 1
- Own home: 1
- Outside porch: 1
- Probation hearing: 1
- Drug screening: 1
- Folding clothes: 1
- Hospital: 1
- Bathroom: 1

Figure S1. *Experience sampling (ESM) questions.*

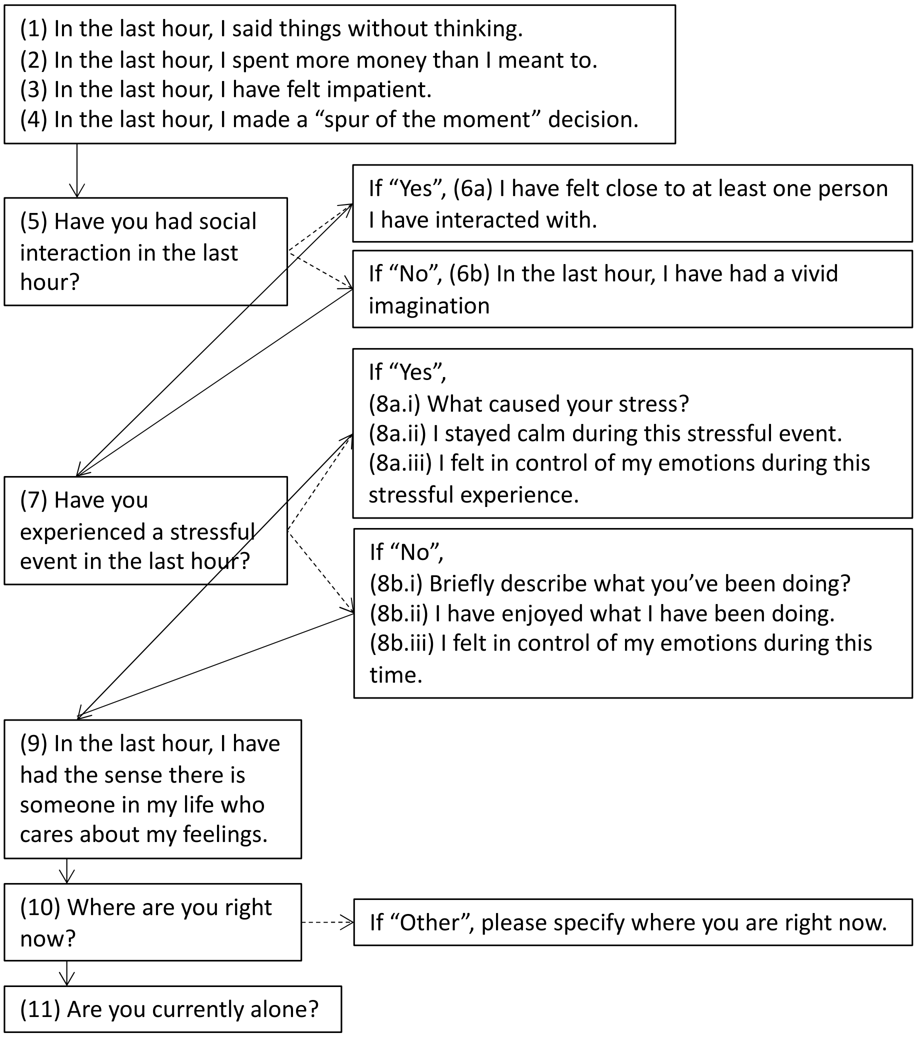

Figure S2. *Ecological momentary intervention (EMI) questions.*

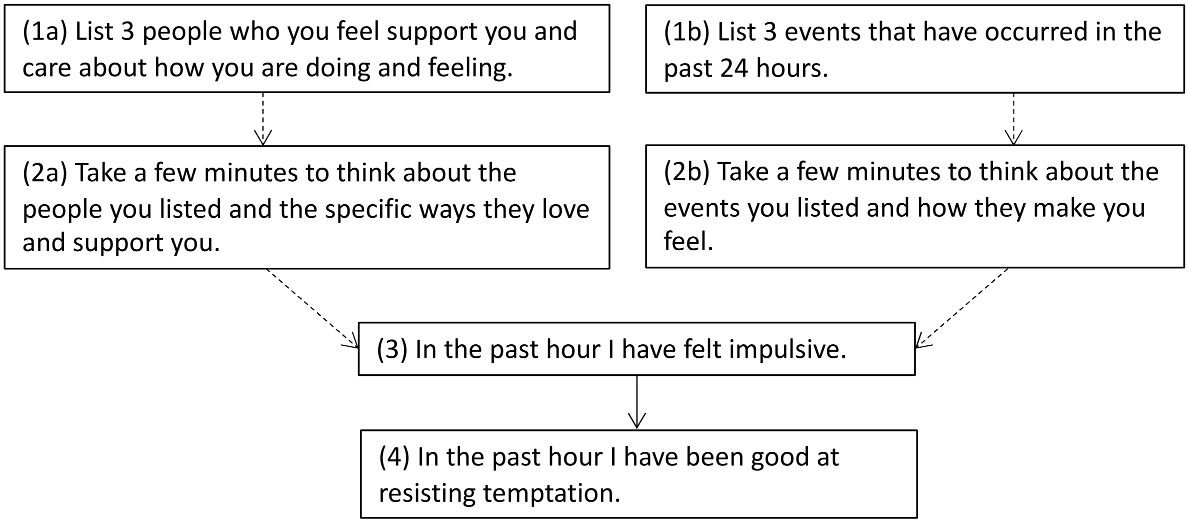

Supplement: Supplementary file 1 — Supplementary Information. [file 41598_2024_53740_MOESM1_ESM.pdf]
